# The role of fibrinolysis in the development of prediabetes-associated coronary heart disease: a focus on the plasminogen activator inhibitor -1 and its potential use as a predictive marker in diet-induced prediabetes

**DOI:** 10.3389/fnut.2023.1256427

**Published:** 2023-11-02

**Authors:** Nompumelelo Gumede, Andile Khathi

**Affiliations:** Department of Human Physiology, School of Laboratory Medicine and Medical Sciences, College of Health Sciences, University of KwaZulu-Natal, Durban, South Africa

**Keywords:** prediabetes, high-fat-high-carbohydrate diet, cardiovascular diseases, C-reactive protein, biomarker, endothelin-1, plasminogen activator inhibitor-1

## Abstract

**Introduction:**

Type 2 diabetes mellitus (T2DM) is associated with an increased risk of cardiovascular diseases (CVD). However, the onset of T2DM is preceded by prediabetes, which is associated with sedentary lifestyles and consumption of high-calorie diets. Studies have shown that impaired glucose homeostasis creates an environment for developing T2DM-related complications. Using a high-fat-high-carbohydrate diet-induced prediabetes animal model, this study sought to assess the risk factors of coronary heart disease (CHD) in diet-induced prediabetes and identify biomarkers that can be used for early detection of prediabetes-associated CHD.

**Methods:**

Male Sprague Dawley rats were randomly grouped into two groups and were kept on different diets for 20 weeks (*n* = 6 in each group). One group was fed standard rat chow to serve as a non-prediabetes (NPD) control, while the other group consumed a high-fat-high-carbohydrate diet to induce prediabetes (PD). Post induction, the homeostasis model assessment- insulin resistance (HOMA-IR) and glycated haemoglobin (HbA1c) was used to test for insulin resistance. Body weight, mean arterial pressure (MAP), resting heart rate (HR), inflammatory cytokines (C-reactive protein (CRP), tumor necrosis factor (TNF-α), interleukin-6 (IL-6)), lipids (total cholesterol (TC), triglyceride (TG), lipoproteins (HDL, LDL, VLDL)), endothelial function (endothelial nitric oxide (eNOS), endothelin -1 (ET-1)), fibrinolysis (plasminogen activator inhibitor-1 (PAI-1)) were all measured to assess the risk of CHD. All data were expressed as means ± S.E.M. Statistical comparisons were performed with Graph Pad. Instat Software using Student’s two-sided *t*-test. The Pearson correlation coefficient and linear regression were calculated to assess the association. The value of *p* < 0.05 was considered statistically significant.

**Results:**

There was significant insulin resistance accompanied by significantly increased HbA1c and body weight in PD compared to NPD. Simultaneously, there was a significant increase in inflammatory cytokines in PD compared to NPD. This was accompanied by significantly increased TG and VLDL and endothelial dysfunction in PD. The association between HOMA-IR and PAI-1 was insignificantly positive in NPD, whereas a significantly strong positive association was observed in PD.

**Conclusion:**

There is a positive correlation between insulin resistance and PAI-1 during prediabetes; therefore, suggesting that prediabetes increases the risk of developing vascular thrombosis. The current therefore study warrants further investigation on PAI-1 and other markers of fibrinolysis for the early detection of thrombosis and risk of CHD in prediabetes.

## Introduction

Type 2 diabetes mellitus (T2DM)-related complications have been shown to begin during prediabetes, an asymptomatic state of intermediate hyperglycemia where blood glucose levels are above normal but below the threshold of T2DM (1). Prediabetes can be described as having impaired fasting glucose (IFG), impaired glucose tolerance (IGT) as well as glycated haemoglobin (HbA1c) (2). However, the inclusion criteria and cut-off values for the diagnosis of prediabetes vary within organizations such as the American Diabetes Association (ADA), World Health Organization (WHO) and the International Diabetes Federation (IDF) and this affects the prevalence and incidence data for this condition (3). The global prevalence of prediabetes in 20-79-year-olds defined by IGT in 2021 was 9.1% (464 million) and is projected to reach 10.0% (638 million) in 2045. When defined by IFG, the prevalence was 5.8% (298 million) and is projected to reach 6.5% (414 million) in 2045 (4). Shockingly, it is reported that the prevalence of prediabetes is 13.9 and 24.6% in children (4–9.9 years) and adolescents (10–17.9 years), respectively and it is increased by the class of obesity (5). The rising burden of prediabetes is postulated to be due to chronic consumption of unhealthy diets, thus leading to obesity and insulin resistance (6, 7). Impaired glucose handling due to insulin resistance produces hyperglycemia, a conducive environment for developing cardiovascular diseases (CVD) (8). Prediabetes is associated with an increased risk of composite CVD and all-cause mortality (8). Hyperglycemia increases diacylglycerol and protein kinase C (PKC) activation in vascular cells. This is associated with inflammation through the activation of nuclear factor κ-В(NF-κв) (9). Activation of NF-Κв stimulates the expression and release of inflammatory cytokines, which injure the vascular endothelium (9). Monocytes infiltrate the site of injury and become macrophages (10). Macrophages engulf activated LDL and become foam cells, which form a fatty streak and ultimately an atherosclerotic plug (10–14). Rupture of the atherosclerotic plug may cause acute vascular infarction (11). Hyperglycaemia, as shown in T2DM, also results in the upregulation of endothelin 1 (ET-1) while reducing nitric oxide (NO) secretion, thus creating an imbalance between endothelial vasoconstrictors and vasodilators (15). Macrophages and vascular smooth muscle cells further increase ET-1 as they also secret ET-1 under hyperglycaemic conditions. This favors vasoconstriction, increased blood pressure and abnormal blood flow (16, 17). Alternatively, fibrinolysis is impaired in T2DM (18, 19). Plasminogen activator inhibitor (PAI-1), which plays a regulatory role in fibrinolysis, is elevated in the arterial wall of diabetic patients (10, 20, 21). An increase in PAI-1 levels causes coronary thrombosis and it isassociated with an increased risk of CHD (22). Assessing the level of PAI-1 during prediabetes, a reversible condition, could help predict the onset or progression of CHD. Using an HFHC diet-induced prediabetes animal developed in our laboratory, previous studies have demonstrated that some T2DM complications begin during prediabetes (23–26). Biomarkers are helpful in predicting a disease’s onset and identifying its presence when fully developed (27). Therefore, there is a need to assess CHD biomarkers during prediabetes for early intervention and prevention. This study aimed to determine the risk factors of CHD in diet-induced prediabetes and identify biomarkers that can be used for the early detection of prediabetes-associated CHD.

## Materials and methods

### Animals

Male Sprague–Dawley rats (150–180 g) were obtained from the biomedical research unit (BRU), University of KwaZulu Natal (UKZN) and kept under standard experimental conditions at room temperature (22±2°*C*), humidity (55 ± 5%), and 12 h day:12 h night cycle. The animals consumed a standard rat chow (Meadow Feeds, South Africa) and water *ad libitum* for 2 weeks to acclimatize before being exposed to an experimental diet (high-fat, high carbohydrate). The high-fat high carbohydrate (HFHC) is composed of carbohydrates (55%kcal/g), fats (30%kcal/g), and proteins (15%kcal/g) as previously described (26, 28). All experimental procedures were conducted in line with the ARRIVE guidelines and according to the ethics and animal care guidelines of the Animal Research Ethics (AREC) Committee of the University of KwaZulu Natal, Durban, South Africa (Ethics No: AREC024/018D).

### Experimental design

After 2 weeks of acclimatization, the animals were grouped into a normal diet group (*n* = 6) and a high-fat high carbohydrate diet group (*n* = 6). The normal diet group was fed a standard rat chow and water *ad libitum.* The high-fat high carbohydrate (HFHC) group was fed an HFHC diet and water supplemented with fructose (15%) for 20 weeks to induce prediabetes. After 20 weeks, the animals were all tested for prediabetes using the American Diabetes Association (ADA) criteria for diagnosis of prediabetes. The ADA criteria involve the use of HbA1c, which has several advantages compared to using FBG and OGTT. Animals with fasting blood glucose (FBG) concentrations of 5.60–7.10 mmol/L, oral glucose tolerance test (OGTT) 2 h glucose concentration of 7.1–11.1 mmol/L and glycated haemoglobin (Hb1Ac) concentration of 5.7–6.4% were considered prediabetic. FBG was determined after a 12 h fasting period using the tail-prick method and measured using a One-Touch select glucometer (Lifescan, Malta, United Kingdom). This was also recorded as time 0 for OGTT. OGTT was conducted as per laboratory-established protocol (29–31). Glucose (0.86 g/kg, p.o.) was loaded into the animals via oral gavage (18-gauge gavage needle, 38 mm long curved with 21/4 mm ball end). Glucose concentrations were measured at 15-, 30-, 60-, and 120-min following glucose loading. HbAc was measured using an ELISA kit. The body weights of all animals were measured at the end of the induction period. At the end of the 20 weeks, the animals kept on a normal diet were found to be non-prediabetic and were termed the non-prediabetes group (NPD), while those kept on an HFHC diet were found to be prediabetic and were termed the prediabetic group (PD).

### The homeostasis model assessment

At the end of the induction period, the Homeostasis Model assessment was used to measure HOMA-IR to assess insulin resistance using the HOMA2 Calculator v2.2.3 program (32). In the homeostasis model assessment (HOMA), insulin resistance is expressed as HOMA-IR value <1.0 = insulin sensitive, > 1.9 = early insulin resistance, > 2.9 = significant insulin resistance (32).

### Blood pressure and heart rate

Blood pressure and heart rate were measured using the non-invasive MRBP IITC Model 31, Life Sciences multichannel tail-cuff blood pressure system (Life Sciences, Woodland Hills, CA), as previously described (28). Briefly, the animals were placed in a restrainer (3${}^{"}$ ID (75 mm)- 12” length) while the tail was attached to the tail cuff. All the rats in the restrainer were placed in a warming chamber (IITC Model 303sc Animal Test Chamber, Life Sciences, Woodland Hills, CA) maintained at 32°*C*. The blood pressure, as well as the heart rate, were measured by occlusion or deflation of the tail-cuff, which detects alteration of blood flow in the tail artery. An average of 3 measured sessions comprising 15 cycles was used for statistical analysis.

### Blood collection and tissue harvesting

The animals were placed in a gas chamber (BRU, UKZN, South Africa) and anesthetized with 100 mg/kg of Isofor (Safeline Pharmaceuticals Ltd., Roodepoort, South Africa) for 3 min to collect blood samples. Blood samples were collected by cardiac puncture into precooled heparinized containers in an unconscious state. The blood samples were centrifuged (Eppendorf centrifuge 5,403, Germany) at 4°*C*, 503 g for 15 min to collect plasma. The plasma was stored at −80°*C* in a Bio Ultra freezer (Snijers Scientific, Tilburg, Holland). The hearts of all the animals were excised, rinsed with cold standard saline solution, weighed, and snapped frozen in liquid nitrogen before storage in a Bio Ultra freezer at −80°*C* for biochemical analysis (28, 31).

### Biochemical analysis

#### Inflammatory cytokines

Inflammatory cytokines (IL-6, TNF-α and CRP) concentration was measured in plasma using their respective rat sandwich ELISA kits (IL-6: E-EL-R0015) (TNF-α: E-EL-R2856) (CRP: E-EL-R0506) according to the manufacturer’s protocol (Elabscience Biotechnology Co., Ltd., Houston, TXA, United States). Briefly, 100 μL of plasma from NPD and PD was pipetted in duplicate in the micro-ELISA plated precoated with a specific antibody. Then, specific biotinylated detection and Avidin-Horseradish Peroxidase (HRP) conjugate were added successively to each microplate well and incubated. Unbound components were washed away. The substrate solution was then added to each well. Only those wells containing rat IL-6/TNF-6/CRP, biotinylated detection antibody and Avidin-HRP conjugated appeared blue.

The enzyme-substrate reaction was terminated by adding a stop solution and the color turned yellow. The optical density (OD) was read spectrophotometrically at a wavelength of 450 nm (Spectrostar Nano spectrophotometer) (BMG Labtech, Ortenburg, LGBW Germany). The OD values of each well were averaged, and the average of the blank was subtracted. The concentration of the samples was interpolated from the standard curve, normality test, descriptive statistics and unpaired *t*-test was performed on Graph Pad Instat Software (version 8.00, GraphPad Software, Inc., San Diego, California, United States).

#### Lipid profile

The total plasma cholesterol (TC), high-density lipoprotein (HDL) cholesterol and triglycerides (TG) were analyzed using colorimetric assays according to the instructions from the manufacturer (TC: E-BC-K109-S) (HDL: E-BC-K222-S) (TG: E-BC-K261-S) (Elabscience Biotechnology Co., Ltd., Houston, TXA, United States). The optical density values were measured via a Spectrostar Nano spectrophotometer (BMG Labtech, Ortenburg, LGBW Germany). Very low-density lipoprotein (VLDL) and low-density lipoprotein (LDL) cholesterol were calculated using Friedewald’s formula: VLDL cholesterol = TG×0.2 and LDL cholesterol = TC− (VLDL cholesterol + HDL cholesterol) (33).

#### Endothelial function

The endothelial function was evaluated from plasma by determining endothelial nitric oxide synthase (eNOS) and endothelin-1 (ET-1) concentration using specific sandwich ELISA kits (eNOS: E-EL-R0367) (ET-1: E-EL-R1458) as per the manufacturer’s protocol (Elabscience Biotechnology Co., Ltd., Houston, TX, United States). Briefly, 100 μL of plasma from NPD and PD was added in duplicate in the micro-ELISA plated precoated with a specific antibody. Then, specific biotinylated detection and Avidin-Horseradish Peroxidase (HRP) conjugate were pipetted successively to each microplate well and incubated. Unbound components were washed away. The substrate solution was then added to each well. Only those wells that contained rat ET-1/eNOS, biotinylated detection antibody and Avidin-HRP conjugated appeared blue in color. The enzyme-substrate reaction was terminated by adding a stop solution and the color turned yellow. The optical density (OD) was read spectrophotometrically at a wavelength of 450 nm (Spectrostar Nano spectrophotometer) (BMG Labtech, Ortenburg, LGBW Germany). The OD values of each well were averaged, and the average of the blank was subtracted. The concentration of the samples was interpolated from the standard curve, normality test, descriptive statistics and unpaired *t*-test was performed on Graph Pad Instat Software (version 8.00, GraphPad Software, Inc., San Diego, California, United States).

### Fibrinolysis

Fibrinolysis was evaluated from the plasma using rat PAI-1 sandwich ELISA (ab201283) according to the manufacturer’s protocol (Abcam Inc., Cambridge, United Kingdom). Briefly, 50 μL of plasma from the NPD and PD samples was diluted in a 1:4 ratio with a Sample Diluent NS. The samples were pipetted in duplicates into separate wells in the micro-ELISA plate. 50 μL of antibody cocktail was pipetted into each well. The plate was then sealed and incubated for 1 h at room temperature on a plate shaker set at 400 rpm (Titramax 1,000, Heidolph Instruments company, Schwabach, Germany). The plate was then washed three times. A 100 μL TMB solution was pipetted into each well and incubated for 10 min in the dark on a plate shaker set at 400 rpm. A 100 μL stop solution was added, and the plate was placed on a plate shaker for 1 min to mix. The OD values were read at the endpoint at a wavelength of 450 nm (Spectrostar Nano spectrophotometer) (BMG Labtech, Ortenburg, LGBW Germany). The OD values of each well were averaged, and the average of the blank was subtracted. The concentration of the samples was interpolated from the standard curve. The concentrations were multiplied by four, the dilution factor. Normality test, descriptive statistics and unpaired *t*-test were performed on Graph Pad Instat Software (version 8.00, GraphPad Software, Inc., San Diego, California, United States).

### Statistical analysis

All data were expressed as means ± S.E.M. Statistical comparisons were performed with Graph Pad Instat Software (version 8.00, GraphPad Software, Inc., San Diego, California, United States) using Student’s unpaired two-sided *t*-test. Pearson correlation coefficient and linear regression were calculated to assess the relationship between HOMA-IR and CRP, HOMA-IR and PAI-1, HbA1c and CRP, as well as HbA1c and PAI-1. A value of *p* < 0.05 was considered statistically significant.

## Results

### HOMA-IR

At the end of the prediabetes induction period (20 weeks), body weights, fasting blood glucose (FBG), plasma insulin and insulin resistance were assessed in the NPD and PD groups. Table 1 shows a significant increase (*p* < 0.0001) in body weights, FBG, plasma insulin and HOMA-IR in the PD group compared to the NPD group. The mean FBG concentration (6.72 ± 0.12 mmol/L) in the PD group was within the ADA prediabetes diagnosis FBG range (5.60–7.10 mmol/L), whereas, for the NPD group, it was below (4.40 ± 0.20 mmol/L). The HOMA-IR value for the NPD was within the insulin sensitivity range (<1.0), whereas it was significantly higher for the PD group and within the insulin resistance range.

**Table 1:** Fasting blood glucose, plasma insulin, and HOMA-IR value between NPD and PD.

NPD		PD	
			*p* value
Body weights (g)	408.60 ± 8.95	622.8 ± 11.46****	<0.0001
Fasting plasma glucose (FBG) (mmol/L)	4.40 ± 0.20	6. 72 ± 0.12****	<0.0001
Plasma insulin (ng/mL)	3.47 ± 0.13	10.87 ± 0.06****	<0.0001
HOMA-IR value	0.68 ± 0.52	3.25 ± 0.06****	<0.0001

****indicates significance *p* = 0.0001.

### Glycated haemoglobin

**Figure 1:**
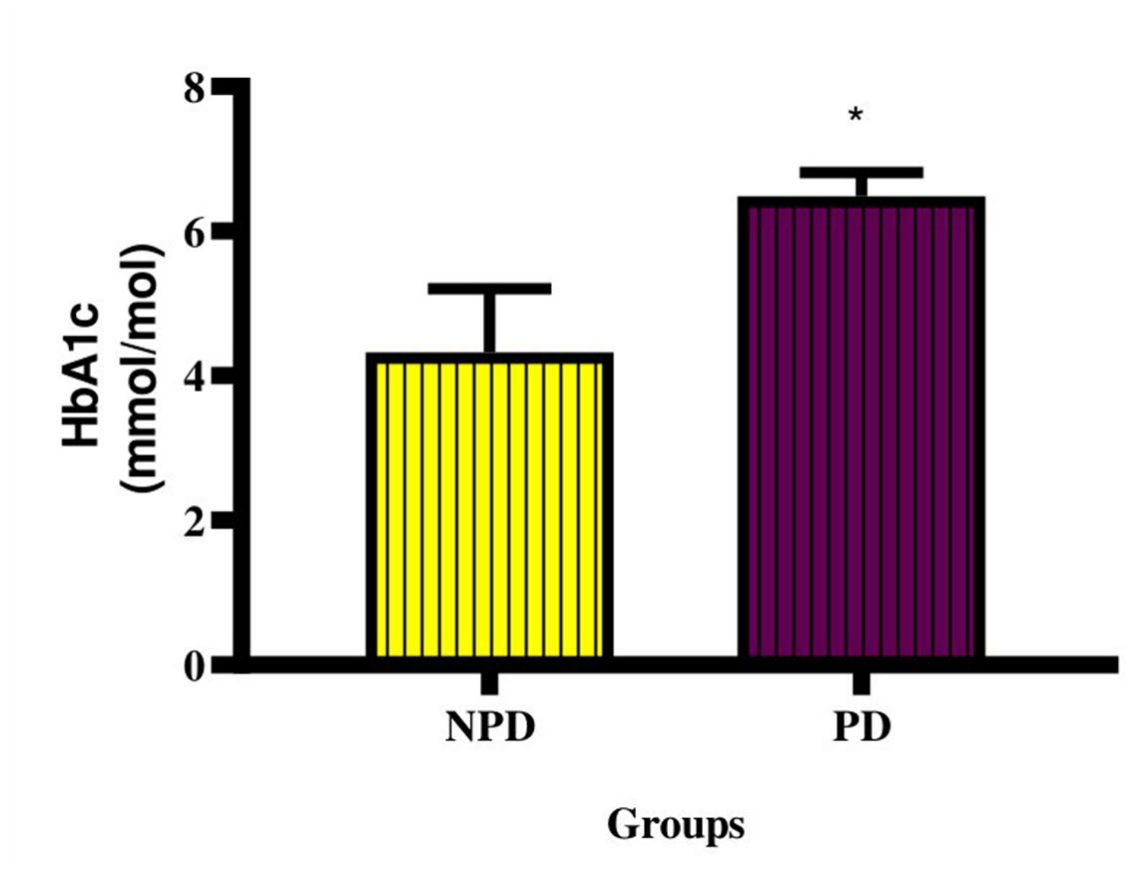
Concentration of HbA1c between the NPD and PD groups. Values are presented as mean ± SEM (*n* = 6 in each group). * Indicates *p* = 0.0443. NPD, non-prediabetes; PD, prediabetes; HbA1c, glycated haemoglobin.

The concentration of HbA1c was measured at the end of the prediabetes induction period between the NPD and PD groups. Figure 1 shows a significant increase (*p* = 0.0443) in the concentration of HbA1c in the PD group compared to the NPD group. The mean HbA1c concentration (6.49 ± 0.32 mmol/mol) of the NPD group was within the prediabetes HbA1c diagnosis range (5.70–7.10%), whereas the mean HbA1c concentration (4.32 ± 0.89 mmol/mol) of the NPD group was below the range.

### Mean arterial pressure and resting heart rate measurement

**Figure 2:**
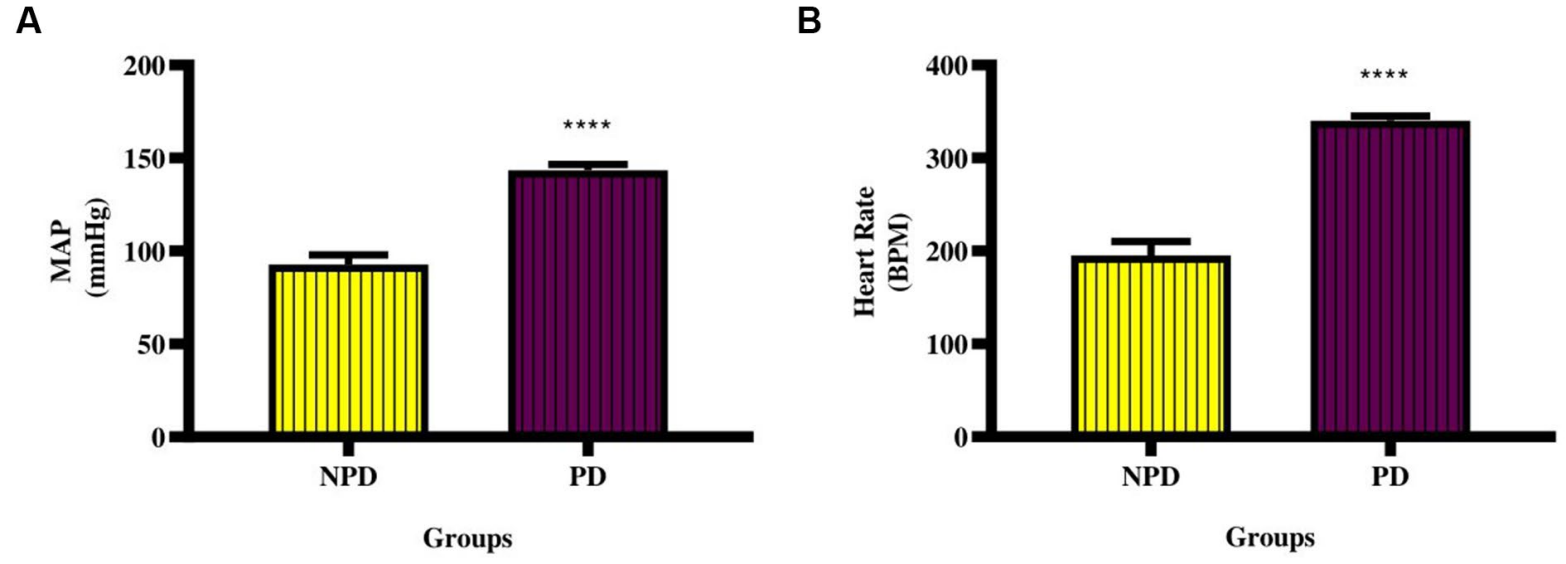
Mean arterial pressure and resting heart rate between the NPD and PD. Values are presented as mean ± SEM (*n* = 6 in each group). **(A)** ****indicates *p* < 0.0001; **(B)** ****indicates *p* < 0.0001 NPD, non-prediabetic; PD, prediabetes; MAP, mean arterial pressure; HR, resting heart rate.

The MAP and resting HR were measured at week 20 between the NPD and PD groups. Figure 2A shows a significant increase (*p* < 0.0001) in MAP (NPD: 92.80 ± 5.14 mmHg; PD: 143.40 ± 3.20 mmHg) and Figure 2B shows a significant increase (*p* < 0.0001) in resting HR (NPD: 195.0 ± 15.07 BPM; PD: 340.30 ± 4.77 BPM) between the NDP and the PD group.

### Inflammatory cytokines

**Figure 3:**
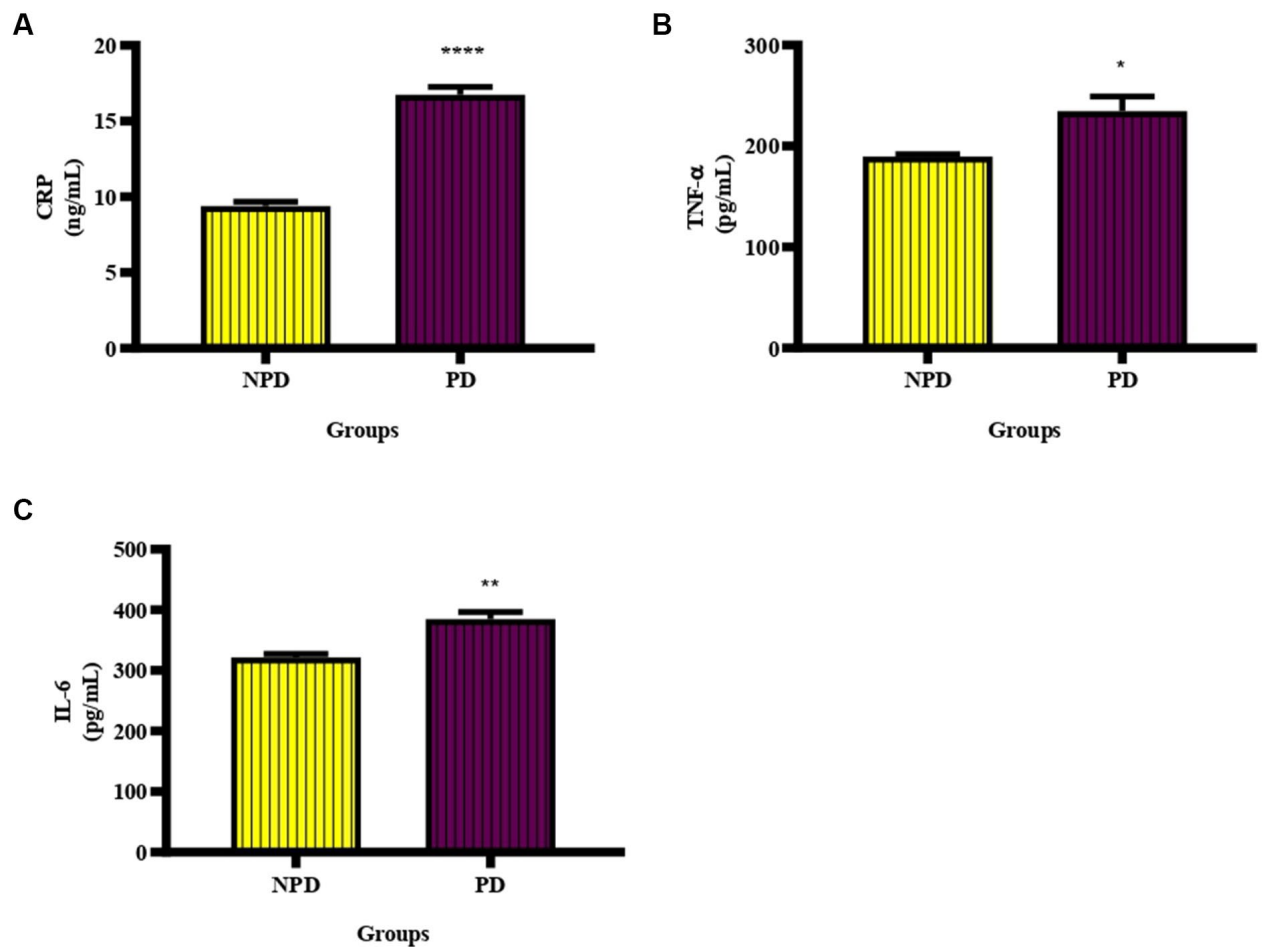
The CRP, TNF- α and IL-6 concentration between the NPD and PD groups. Values are presented as mean ± SEM (*n* = 6 in each group). **(A)** ****indicates *p* < 0.0001, **(B)** *indicates *p* = 0.0369, **(C)** **indicates *p* = 0.0078. NPD, non-prediabetic; PD, prediabetic; CRP, C-reactive protein, TNF-α, tumor necrosis factor alpha; IL-6, interleukin 6.

The CRP, TNF-α and IL-6 concentration was measured in plasma at week 20 between the NPD and PD groups. Figure 3A shows a significant increase (*p* < 0.0001) in CRP (NPD: 9.39 ± 0.29 ng/mL; PD: 16.74 ± 0.53 ng/mL), Figure 3B shows a significant increase (*p* = 0.0369) in TNF-α (NPD: 189.60 ± 2.34 pg./mL; PD: 234.60 ± 14.42 pg./mL), and Figure 3C shows a significant increase (*p* = 0.0078) in IL-6 (NPD: 321.60 ± 5.60 pg./mL; PD: 384.90 ± 11.50 pg./mL) in the PD group compared to the NPD group.

### Lipid profile

The TG, TC and HDL concentration between the NPD and PD groups was measured in plasma at week 20. The concentration of LDL and VLDL was calculated using Friedewald’s formula. Table 2 shows a significant increase in the concentration of TG and VLDL in the PD compared to the NPD group, while there was a significant decrease in the concentration of LDL in the PD compared to the NPD.

**Table 2:** Lipid concentration between NPD and PD groups.

	NPD	PD	
			*p* value
TG	1,37 ± 0.16	6.54 ± 0.04****	<0.0001
TC	4.00 ± 0.05	4.05 ± 0.07	0.6041
HDL	0.63 ± 0.10	0.73 ± 0.13	0.3611
LDL	3.11 ± 0.07	1.98 ± 017***	0.0004
VLDL	0.27 ± 0.03	1.31 ± 0.01****	<0.0001

***, ****corresponds to the *p*-values given in the table.

### Endothelial function

**Figure 4:**
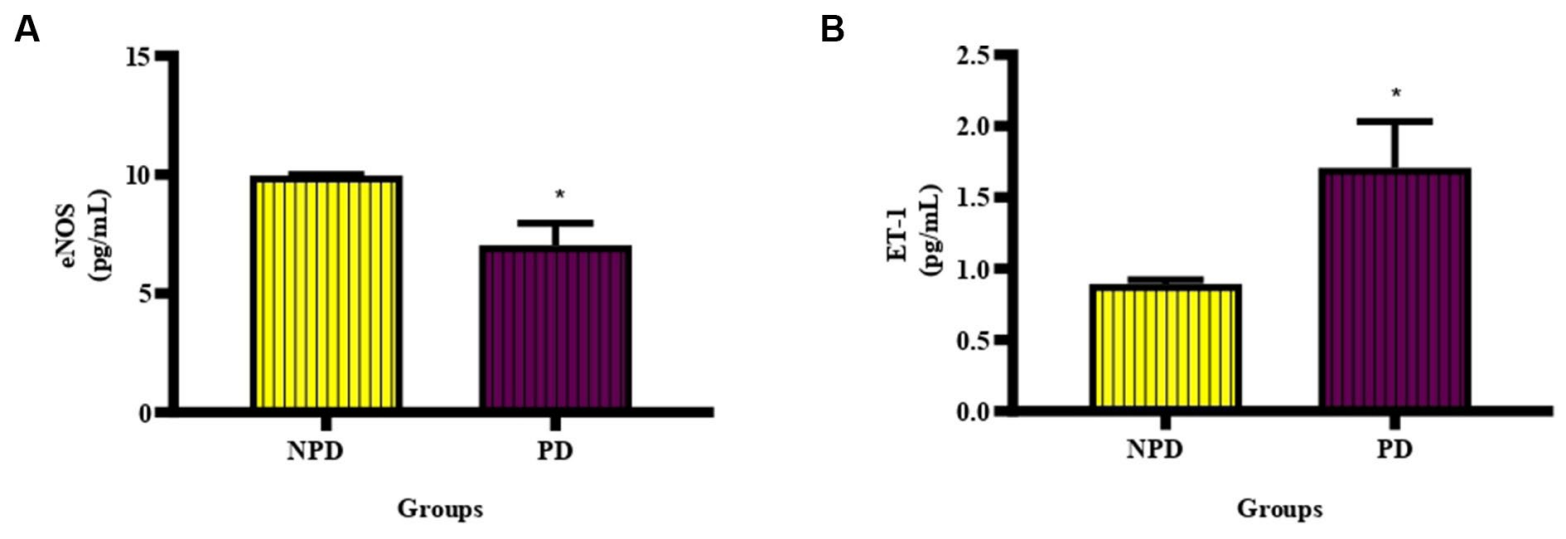
The concentration of eNOS and ET-1 between the NPD and PD groups. Values are presented as mean ± SEM (*n* = 6 in each group). **(A)** *Indicates *p* = 0.0338, **(B)** *indicates *p* = 0.0463; NPD, non-prediabetic; PD, prediabetes; eNOS, endothelial nitric oxide synthase; ET-1, endothelin-1.

The concentration of eNOS and ET-1 was measured in plasma at week 20 between the NPD and PD groups. In Figure 4A shows a significant decrease (*p* = 0.0338) in eNOS (NPD: 9.97 ± 0.03 pg./mL; PD: 7.04 ± 0.93 pg./mL) and Figure 4B shows a significant increase (*p* = 0.0463) in ET-1 (NPD: 0.89 ± 0.03 pg./mL; PD: 1.71 ± 0.32 pg./mL) in the PD group compared to the NPD group.

### Fibrinolysis

**Figure 5:**
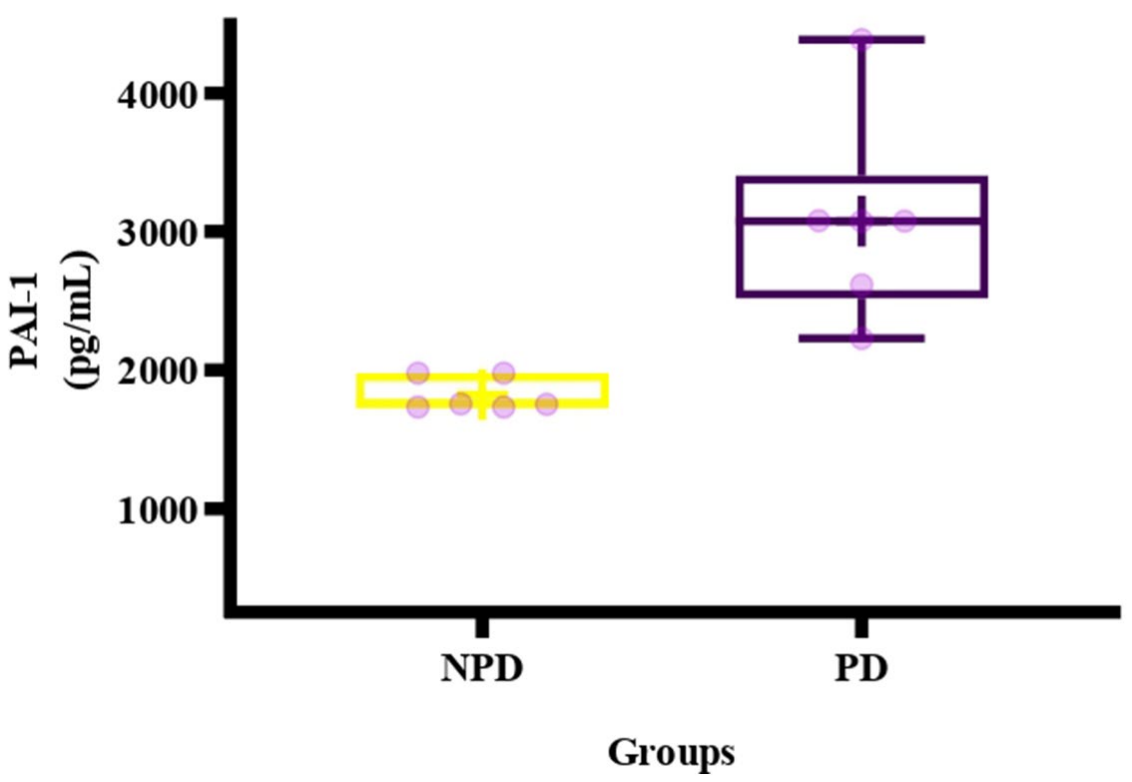
Box plot of the concentration of PAI-1 in the NPD and PD groups. The top of the box represents the 75th percentile, the bottom of the box represents the 25th percentile, and the line in the middle of the box represents the 50th percentile. The whiskers represent the highest and lowest values. + in the middle of the box shows the mean. NPD, non-prediabetes; PD, prediabetes; PAI-1, plasminogen activator inhibitor-1.

The concentration of PAI-1 was analyzed in plasma at the end of week 20 between the NPD and PD groups. Figure 5 shows a box and whisker plot for the concentration of PAI-1 between the NPD and PD groups. The mean concentration of PAI-1 (3077 ± 664.30 pg./mL) in the PD group was higher compared to the NPD group (1821 ± 78.91 pg./mL); however, the increase was insignificant (*p* = 0.1336).

### Correlation and linear regression analysis

Correlation and linear regression between HOMA-IR and CRP, HOMA-1R and PAI-1, between HbA1c and CRP, as well as HbA1c and PAI-1, was calculated between the NPD and PD groups. Table 3 shows an insignificant positive association between HOMA-IR and CRP concentration of the NPD group (*r* = 0.40, *p* = 0.6047) and the PD group (*r* = 0.93, *p* = 0.0682). Regression results depicted that the model predicts a 16% (*R*^2^ = 0.16, *p* = 0.6047) change in CRP in the NPD group compared to an 87% change in the PD group (*R*^2^ = 0.87, *p* = 0.0682). There was an insignificant positive association between HOMA-IR and PAI-1 concentration in the NPD (*r* = 0.05, *p* = 0.9690). In contrast, the PD group had a strong positive and significant association with PAI-1 concentration (*r* = 1, *p* = 0.0227). Regression analysis showed that the model predicts a 0% (*R*^2^ = 0.00, *p* = 0.9690) relationship between HOMA-IR and PAI-1 in the NPD group. In contrast, there was a 99.87% (*R*^2^ = 0.9987, *p* = 0.0227) relationship between HOMA-IR and PAI-1 in the PD group. The association between HbA1c and CRP concentration was positive and insignificant in the NPD (*r* = 0.85, *p* = 0.1472) and PD groups (*r* = 0.09, *p* = 0.9145). Regression analysis showed a 73% (*R*^2^ = 0.73, *p* = 0.1472) and a 10% (*R*^2^ = 0.01, *p* = 0.9145) relationship between HbA1c and CRP in the NPD and PD groups, respectively. The association between HbA1c and the concentration of PAI was insignificant in the NPD group (*r* = 0.38, *p* = 0.7550) and PD group (*r* = 0.76, *p* = 0.4512). Regression analysis showed that the model predicts a 14% (*R*^2^ = 0.14, *p* = 0.7750) and a 58% (*R*^2^ = 0.58, *p* = 0.4512) change in PAI-1 in the NPD and PD groups, respectively.

**Table 3:** Correlation and linear regression analysis between insulin resistance markers with C-reactive protein and plasminogen activator inhibitor -1.

	CRP (ng/mL) vs. HOMA-IR		PAI-1 (pg/mL) vs. HOMA-IR	
	NPD	PD	NPD	PD
Correlation	*r* = 0.40	*r* = 0.93	*r* = 0.05	*r* = 1*
	*p* = 0.6047	*p* = 0.0682	*p* = 0.9690	*p* = 0.0227
Linear regression	*R*^2^ = 0.16	*R*^2^ = 0.87	*R*^2^ = 0.00	*R*^2^ = 1
	y = 1.440*X + 8.383	y = 5.616*X − 1.455	y = 69.93*X + 1766	y = 30374*X − 98880
	*p* = 0.6047	*p* = 0.0682	*p* = 0.9690	*p* = 0.0227*

	CRP (ng/mL) vs. HbA1c(mmol/mol)		PAI-1 (pg/mL) vs. HbA1c (mmol/mol)	
Correlation	*r* = 0.85	*r* = 0.09	*r* = 0.38	*r* = 0.76
	*p* = 0.1472	*p* = 0.9145	*p* = 0.7550	*p* = 0.4512
Linear regression	*R*^2^ = 0.73	*R*^2^ = 0.01	*R*^2^ = 0.14	*R*^2^ = 0.58
	y = 1.057*X + 3.473	y = 0.2418*X + 15.03	y = 153.0*X + 935.3	y = 1975*X − 10824
	*p* = 0.1472	*p* = 0.9145	*p* = 0.7750	*p* = 0.4512

*indicates significance *p* = 0.0227.

## Discussion

Chronic consumption of high-calorie diets is associated with a high prevalence of T2DM and its complications (34). Studies have shown that the complications of T2DM begin during prediabetes (35). Indeed, studies in our laboratory using an HFHC-diet-induced prediabetes model have demonstrated early diabetic nephropathy, HPA axis dysregulation and non-alcoholic fatty liver disease (23, 36). However, the risk factors and biomarkers associated with CVD development have not been assessed. Therefore, this study sought to determine the risk factors of CHD in diet-induced prediabetes as well as identify biomarkers that can be used for the early detection of prediabetes-associated CHD.

Insulin is crucial in stimulating glucose uptake and oxidation in the heart, skeletal muscle, liver and adipose tissue (37). Insulin resistance is the inability of target tissues to respond to insulin due to defects in insulin receptors, thus decreasing insulin-mediated glucose regulation (38). Defects are reported in the IR, IRS, PI3K, and Akt pathways in insulin signaling and GLUT4 expression (38). Obesity, which can occur due to several factors such as overconsumption of high-calorie diets, physical inactivity, genetics and age, has been identified as the leading cause of insulin resistance (39). Indeed, in the present study, animals in the PD group who consumed a high-calorie diet (HFHC diet) became obese, as indicated by a significant increase in body weight compared to the NPD animals that consumed a standard rat chow. Obesity is associated with chronic low-grade inflammation and is responsible for insulin resistance (39).

The role of obesity in insulin resistance is through the release of free fatty acids (FFA) and inflammatory mediators. The binding of FFA to toll-like receptors (TLR) downregulates PI3K (phosphatidylinositol 3 kinase) and Akt, leading to reduced expression of GLUT4 and, subsequently, the response to insulin binding (15). The HOMA-IR index is used to assess insulin resistance (32). The concentration of HbA1c is used to determine the average plasma glucose over an extended period (40). In this study, the PD group’s HOMA-IR value was higher than the NPD group and in the range of significant insulin resistance. The PD’s FBG and plasma insulin concentrations were also significantly elevated compared to the NPD group. These results suggest insulin resistance in PD. Furthermore, the concentration of HbA1c was significantly increased in the PD group compared to the NPD. These results concur with the finding of a study by Luvuno et al., in which chronic consumption of HFHC diet-induced prediabetes evidenced by insulin resistance and increased HbA1c (28).

Inflammation is the normal homeostatic response of the body during infection from pathogens (41). The normal inflammatory response is acute and involves activating immune and non-immune cells (42). Immune and vascular cells produce cytokines such as TNF-α and IL-6 during an inflammatory immune response (43). TNF-α is a proinflammatory cytokine that functions in cytokine expression, tumor necrosis, and neutrophil activation. Conversely, IL-6 functions in cell proliferation and differentiation of B-cells into plasma cells and is also pro-atherogenic (43). Hyperglycemia promotes low-grade inflammation by upregulating NF-kВ (9, 42). Inflammation is one of the mechanisms associated with CVD development in prediabetes and T2DM (44). Increased concentration of inflammatory cytokines causes injury to the vascular endothelium (45). Elevated IL-6 is associated with initiating chronic inflammation (46). Çakar and colleagues reported that CRP has a positive association with e-selectin, a marker of endothelial injury (47). These studies indicate that inflammatory cytokines cause vascular endothelium injury. The damage to the vascular endothelium initiates the process of atherosclerosis, which is the underlying cause of ischaemic heart disease (45, 48). Elevated CRP levels are associated with an increased risk of plaque rupture and vascular thrombosis (49). In this study, intermediate hyperglycaemia caused a significant increase in CRP, TNF-α and IL-6 concentration in the PD compared to the NPD group.

These results coincide with a study by Mzimela et al., in which prediabetes had significantly increased CRP, TNF-α, and IL-6 with increased lymphocytes, neutrophils and monocytes (25). In Table 3, we reported a positive correlation between HbA1c and CRP in the NPD and PD groups and a linear regression that predicts a 73% positive relationship between HbA1c and CRP in the NPD and a 10% relationship in the PD group. InThere was an insignificant positive correlation between HOMA-IR and CRP in the NPD and PD and a linear regression that predicts a 16% relationship between HOMA-IR and CRP in the NDP group and an 87% relationship in the PD group. Veerasak and Warangkana found a significant positive correlation between CRP and HbA1c in obese T2DM individuals with HbA1c concentrations of 7.36 ± 1.23% and 8.77 ± 1.78% (50). The subjects in this study are obese and diabetic. The significant positive correlation could be due to obesity and high HbA1c concentrations. In the current study, the animals were obese, prediabetic and had lower HbA1c concentrations than T2DM. Wang et al. reported an insignificant positive correlation between CRP and HOMA-IR in individuals with prediabetes (51). These results align with the findings of the current study. The result of the present study further demonstrates that the concentration of CRP increases with increasing levels of insulin resistance during prediabetes. This also indicates that prediabetic individuals are at risk of developing CHD and the severity is dependent on the duration of prediabetes and the level of insulin resistance. Prediabetic individuals should be tested for inflammatory biomarkers and be advised on therapeutic or dietary methods to reverse prediabetes or reduce inflammation.

Lipids and lipoproteins are responsible for cholesterol metabolism (52). HDL removes cholesterol from peripheral tissues for excretion through the liver (53). HDL is viewed as anti-atherosclerotic, anti-inflammatory, and anti-thrombogenic and plays a role in vasodilation (53). LDLs and VLDLs transport fat molecules to all peripheral tissues, whereas TGs act as an energy source and transporters of dietary fats (54). Central adiposity is associated with dyslipidaemia and insulin resistance; conversely, insulin resistance increases the production of FFA and TG while decreasing HDL (55). In T2DM, insulin resistance results in abnormal FFA metabolism and elevated hepatic output of TG (56). Insulin resistance increases TG by increasing hepatic VLDLs and low levels of lipoprotein lipase (57). This study showed a significant increase in TG and VLDL in the diet-induced prediabetic animals compared to the NPD group. Interestingly, there was a significant decrease in LDL in the PD group compared to the NPD. Increased concentration of TG and VLDL is reported in previously published papers (31, 58, 59). In a study by Katzke et al. TC, apoB-100 and TG were positively associated with MI. Furthermore, high levels of TG were associated with an increased risk of stroke (52). ApoE (apolipoprotein E) and AIP1(apoptosis signal-regulating 1 interacting protein 1) knockout mice fed a Western-type diet demonstrated elevated inflammatory cytokines (TNF-α, IL-6, IL-10 and IL-12) and lipoproteins (VLDL and LDL). Photomicrographs of the aorta of the ApoE and AIP1 knockout mice showed a greater area of atherosclerosis plague development compared to ApoE knockout mice (60). This study demonstrates the role of inflammation-induced hyperlipidaemia as well as atherosclerosis.

Numerous studies have documented lipids and lipoproteins as risk factors for CVDs, with LDL stated to be a strong predictor of atherosclerosis and CVD development (61–63). However, Hsu et al. recently reported that small dense LDL cholesterol (sdLDL-C) is a stronger CVD risk predictor than LDL. The level of sdLDL-C can detect individuals at low risk based on LDL levels. Furthermore, sdLDL-C is strongly associated with inflammation, thrombosis, haematological biomarkers and prediabetes (64). VLDL was shown to have a stronger association with coronary artery calcification than TG in the Penn Diabetes Heart Study (PDHS) (65, 66). VLDLs are minuscule and, therefore, enter the vascular wall and are trapped in the intima, resulting in the development of atherosclerosis through foam cell formation and low-grade inflammation (67).

The endothelium is the internal layer of the blood vessels, cardiac valves and other body cavities (68). The endothelium plays a vital role in regulating vascular tone, inflammation, and thrombosis by releasing vasoactive substances (68). NO is a potent vasodilator synthesized by eNOS and opposes the vasoconstrictive effects of ET-1 (69). Besides being a vasodilator, NO suppresses leukocyte adhering, platelet activation and PAI-1 expression (70). ET-1 is a potent endothelium-derived vasoconstrictor with pro-fibrotic, pro-oxidative, and proinflammatory properties (71). Insulin resistance contributes to endothelial dysfunction in prediabetes and T2DM (72). Endothelium dysfunction is also attributed to chronic inflammation; conversely, endothelial dysfunction also results in inflammation (70). This study showed a significant increase in inflammatory mediators, which could have caused vascular endothelium dysfunction. Endothelium dysfunction is an early sign of atherosclerosis and a CVD risk factor (70). Hyperglycaemia results in the upregulation of ET-1 while inhibiting eNOS through the activation of PKC (15, 71). It has been reported that ET_A_ and ET_B_ impair insulin-mediated vasorelaxation in the aorta and are improved by administering ET_A_ and ET_B_ antagonists (73). Indeed, in this study, there was a significant decrease in the concentration of eNOS and a significant increase in the concentration of ET-1. Decreased eNOS concentration coincides with previously published papers’ findings (31, 74, 75). Sachidanandam and colleagues reported a significant increase in the concentration of ET-1 in Wistar and Goto-Kakizaki (GK) rats in their study evaluating the differential effects of HFD-induced dyslipidaemia and hyperglycaemia on resistance mesenteric artery structure (76). Sud and Black demonstrated that ET-1 reduces the expression of eNOS and NO secretion in endothelial cells through PKCδ-mediated STAT3 activation (77). This endothelial dysfunction is associated with atherosclerosis and reduced blood vessel diameter (78, 79). This study observed that prediabetes does not only decrease NO synthesis and secretion. However, it also increases the concentration of ET-1. Elevation of ET-1, a potent vasoconstrictor, could be considered a risk factor for CHD.

An intact vascular wall and a balance between procoagulants and anticoagulants maintain blood fluidity (80). An anticoagulant state is maintained by (1) antithrombin, (2) thrombomodulin secreted by an intact endothelium, and (3) fibrinolysis by plasmin (81–83). Fibrin stabilizes the platelet plug at the site of injury in the vessel, thus preventing blood loss (84). When the blood vessel has healed, plasmin is generated by tPA or u-PA to lyse fibrin (84). PAI-1 is the primary inhibitor of tPA and uPA (72). Elevated levels of PAI-1 are associated with coronary thrombosis and have been reported to be associated with an increased risk of MI (85). The concentration of PAI-1 is increased by insulin resistance, endothelium dysfunction and inflammation (86). Insulin resistance, endothelial dysfunction and inflammation have been reported in the is study. In the Insulin Resistance Atherosclerosis Study (IRAS), the odds ratio of nondiabetic subjects with risks of developing diabetes correlated with an increase in PAI-1 and CRP (87). A decrease in NO, such as in endothelial dysfunction, has been linked with platelet aggregation and increased PAI-1 (70, 88). In the present study, there was an increase in the concentration of PAI-1 in the PD compared to the NPD, but it was not statistically different. We noted a vast difference in the SEMs, with the PD group having a high SEM. The spread of data points in the PD could be the reason for non-significance. These findings differ from results published by Sundaram et al., in which a high-fat (HF) diet supplemented with PUFA significantly increased the concentration of PAI-1 in plasma in mice compared to a low-fat (LF) diet (89). Most studies evaluate PAI-1 in obese, PAI-1 knockout animal models or obese human subjects (90–93). The increase in adipose tissue could be why these studies reported statistically significant results, as the adipose tissue releases PAI-1 in parallel with increased fat mass (90). Though there was an increase in BMI and insulin resistance in prediabetes, we speculate that it was insufficient to increase PAI-1 concentration significantly. Table 3 shows an insignificant positive correlation between HbA1c and PAI-1 in the NPD and PD groups. Table 3 also shows a 14% and a 58% relationship between HbA1c and PAI-1 in the NPD and PD groups, respectively. There was a significant, positive correlation between HOMA-IR and PAI-1 in the PD group but insignificant in the NPD. Linear regression analysis showed a 0% relationship between HOMA-IR and PAI-1 in the NPD group and a 99.87% relationship in the PD group. Elevated concentration of PAI-1 has been reported in prediabetic individuals classified according to the ADA criteria (94). Pallela and colleagues recently reported a significant positive correlation between PAI-1 and HbA1c in T2DM individuals with HbA1c < 7% and those with HbA1c ≥ 7% (95). These results concur with those published by Nkansah and colleagues, who reported a significant positive correlation between PAI-1 and HbA1c in T2DM individuals with HbA1c < 7% and those with HbA1c ≥ 7% (96). These studies were conducted in a large population of T2DM individuals with a long history of diabetes. This could be why they found evidence of statistical difference, whereas we did not. Prediabetes was induced over a period of 20 weeks.

Overt metabolic changes were not yet present; the compensatory mechanism is not yet dysfunctional. In newly diagnosed (≤ 3 years) T2DM individuals, Kahn and colleagues reported a significant positive association between the concentration of PAI-1 and HOMA-IR and argued that changes in adipose and fibrinolysis markers are due to adipose tissue and not insulin resistance in newly diagnosed T2DM (97). In a study evaluating the difference in PAI-1 levels in metabolically healthy and unhealthy obese individuals, the concentration of PAI-1 had a significant positive correlation with HOMA-IR (98). These studies show a significant correlation between PAI-1 and HOMA-IR in T2DM and human studies. According to our knowledge, it is the first time that a positive and significant correlation between PAI-1 and HOMA-IR has been reported in diet-induced prediabetes. These results indicate that PAI-1 levels are elevated in relation to insulin resistance in prediabetes. Therefore, assessing the levels of PAI-1 during prediabetes could potentially help in the early detection of CHD risks.

A systolic blood pressure (SBP) of 130–139 mmHg and a diastolic blood pressure (DBP) of 85–89 mmHg is considered high normal, whereas that of SBP≥180 and DBP≥110 is grade 3 hypertension and an increased risk of CVD (99). Vascular inflammation has been linked with hypertension through CRP, directly decreasing the expression of eNOS (100, 101). Impaired NO-mediated vasodilation increases BP (102). Elevated ET-1 has been shown to reduce ANP-mediated vasorelaxation in the aorta through cGMP reduction, thus increasing BP (103, 104). The current study showed a significant increase in MAP and resting HR. These results align with the results published by Luvuno et al. (105) and Akilen et al. (106). Increased BP if left untreated overt to hypertension (107). Furthermore, hypertension is a significant risk factor for atherosclerosis in coronary blood vessels (107).

## Conclusion

Chronic consumption of a HFHC diet induced prediabetes through obesity and insulin resistance which in turn led to inflammation and endothelial dysfunction which are risk factors of CHD. There is a positive correlation between insulin resistance and PAI-1 as shown by HOMA-IR and PAI-1 therefore suggesting that prediabetes increases the risk of developing vascular thrombosis. While there are several other markers of fibrinolysis, this study showed PAI-1 has potential for use as a biomarker in the development of prediabetes-associated CHD. The current therefore study warrants further investigation on PAI-1 and other markers of fibrinolysis for the early detection of thrombosis and risk of CHD in prediabetes.

## Limitations

Biochemical analyses were done soon after prediabetes induction; therefore, the study is based on changes that occur early on in prediabetes. We believe extending the duration and increasing the number of animals per group could give a better view of the biomarkers. The analytes measured in the study were not assessed directly in the heart. Therefore, it is unknown if the change in biomarkers is also present in the heart. Only PAI-1 was used to determine fibrinolysis; we could have added more indicators of fibrinolysis.

## Future recommendations

Future studies should delve into changes in cardiac physiology, histology and therapy to determine if the cardiac function and structure are compromised in prediabetes and if the currently available treatment is beneficial in preventing cardiac injury and progression to overt CHD.

## Data availability statement

The raw data supporting the conclusions of this article will be made available by the authors, without undue reservation.

## Ethics statement

The animal study was approved by Animal Research Ethics (AREC) Committee of the University of KwaZulu Natal, Durban, South Africa (Ethics No: AREC024/018D). The study was conducted in accordance with the local legislation and institutional requirements.

## Author contributions

NG: Formal analysis, Investigation, Writing – original draft. AK: Conceptualization, Formal analysis, Funding acquisition, Methodology, Supervision, Writing – review & editing.

## References

[1] Schlesinger S Neuenschwander M Barbaresko J Lang A Maalmi H Rathmann W. Prediabetes and risk of mortality, diabetes-related complications and comorbidities: umbrella review of meta-analyses of prospective studies. Diabetologia. (2022) 65:275–85. doi: 10.1007/s00125-021-05592-3, PMID: 34718834 PMC8741660

[2] Echouffo-Tcheugui JB Selvin E. Prediabetes and what it means: the epidemiological evidence. Annu Rev Public Health. (2021) 42:59–77. doi: 10.1146/annurev-publhealth-090419-102644, PMID: 33355476 PMC8026645

[3] Hostalek U. Global epidemiology of prediabetes – present and future perspectives. Clin Diabetes Endocrinol. (2019) 5:5. doi: 10.1186/s40842-019-0080-0, PMID: 31086677 PMC6507173

[4] Rooney MR Fang M Ogurtsova K Ozkan B Echouffo-Tcheugui JB Boyko EJ. Global prevalence of prediabetes. Diabetes Care. (2023) 46:1388–94. doi: 10.2337/dc22-2376, PMID: 37196350 PMC10442190

[5] Pedicelli S Fintini D Ravà L Inzaghi E Deodati A Spreghini MR. Prevalence of prediabetes in children and adolescents by class of obesity. Pediatr Obes. (2022) 17:e12900. doi: 10.1111/ijpo.12900, PMID: 35144324 PMC9286831

[6] Madlala SS Hill J Kunneke E Kengne AP Peer N Faber M. Dietary diversity and its association with nutritional status, cardiometabolic risk factors and food choices of adults at risk for type 2 diabetes mellitus in Cape Town, South Africa. Nutrients. (2022) 14:3191. doi: 10.3390/nu14153191, PMID: 35956367 PMC9370540

[7] Sharma MD Garber AJ. What is the best treatment for prediabetes? Curr Diab Rep. (2009) 9:335–41. doi: 10.1007/s11892-009-0053-2 19793502

[8] Huang Y Cai X Mai W Li M Hu Y. Association between prediabetes and risk of cardiovascular disease and all cause mortality: systematic review and meta-analysis. BMJ. (2016) 355:i5953. doi: 10.1136/bmj.i5953 27881363 PMC5121106

[9] Chait A Bornfeldt KE. Diabetes and atherosclerosis: is there a role for hyperglycemia? J Lipid Res. (2009) 50:S335–9. doi: 10.1194/jlr.R800059-JLR200, PMID: 19029122 PMC2674740

[10] Cheung BM Li C. Diabetes and hypertension: is there a common metabolic pathway? Curr Atheroscler Rep. (2012) 14:160–6. doi: 10.1007/s11883-012-0227-2, PMID: 22281657 PMC3314178

[11] Fowler MJ. Microvascular and macrovascular complications of diabetes. Clin Diabetes. (2011) 29:116–22. doi: 10.2337/diaclin.29.3.116

[12] Forbes JM Cooper ME. Mechanisms of diabetic complications. Physiol Rev. (2013) 93:137–88. doi: 10.1152/physrev.00045.2011 23303908

[13] Park YM Febbraio M Silverstein RL. CD36 modulates migration of mouse and human macrophages in response to oxidized LDL and may contribute to macrophage trapping in the arterial intima. J Clin Invest. (2009) 119:136–45. doi: 10.1172/JCI35535, PMID: 19065049 PMC2613464

[14] Curtiss LK. Reversing atherosclerosis? N Engl J Med. (2009) 360:1144–6. doi: 10.1056/NEJMcibr0810383 19279347

[15] Huang D Refaat M Mohammedi K Jayyousi A Al Suwaidi J Abi KC. Macrovascular complications in patients with diabetes and prediabetes. Biomed Res Int. (2017) 2017:1–9. doi: 10.1155/2017/7839101 PMC5697393 29238721

[16] Li W Abdul Y Ward R Ergul A. Endothelin and diabetic complications: a brain-centric view. Physiol Res. (2018) 67:S83–94. doi: 10.33549/physiolres.933833, PMID: 29947530

[17] Banting Lecture BM. The pathobiology of diabetic complications. A unifying mechanism. Diabetes. (2004) 54:1615–25. doi: 10.2337/diabetes.54.6.1615 15919781

[18] Undas A Wiek I Stêpień E Zmudka K Tracz W. Hyperglycemia is associated with enhanced thrombin formation, platelet activation, and fibrin clot resistance to lysis in patients with acute coronary syndrome. Diabetes Care. (2008) 31:1590–5. doi: 10.2337/dc08-0282, PMID: 18487475 PMC2494657

[19] Dunn E Philippou H Ariëns R Grant P. Molecular mechanisms involved in the resistance of fibrin to clot lysis by plasmin in subjects with type 2 diabetes mellitus. Diabetologia. (2006) 49:1071–80. doi: 10.1007/s00125-006-0197-4, PMID: 16538489

[20] Pandolfi A Cetrullo D Polishuck R Alberta M Calafiore A Pellegrini G. Plasminogen activator inhibitor type 1 is increased in the arterial wall of type II diabetic subjects. Arterioscler Thromb Vasc Biol. (2001) 21:1378–82. doi: 10.1161/hq0801.093667 11498469

[21] Soares AL Sousa MO Fernandes A Carvalho MG. Hemostatic changes in patients with type 2 diabetes mellitus. Rev Bras Hematol Hemoter. (2010) 32:482–8. doi: 10.1590/S1516-84842010000600013

[22] Tofler GH Massaro J O'Donnell CJ Wilson PWF Vasan RS Sutherland PA. Plasminogen activator inhibitor and the risk of cardiovascular disease: the Framingham heart study. Thromb Res. (2016) 140:30–5. doi: 10.1016/j.thromres.2016.02.002, PMID: 26896607 PMC5722217

[23] Gamede M Mabuza L Ngubane P Khathi A. Preventing the onset of diabetes-induced chronic kidney disease during prediabetes: the effects of oleanolic acid on selected markers of chronic kidney disease in a diet-induced prediabetic rat model. Biomed Pharmacother. (2021) 139:111570. doi: 10.1016/j.biopha.2021.111570, PMID: 33932738

[24] Mkhize BC Mosili P Ngubane PS Sibiya NH Khathi A. Dietinduced prediabetes: Effects on the activity of the renin-angiotensin-aldosterone system in selected organs. Journal of Diabetes Investigation. (2022) 13:768–780. doi: 10.1111/jdi.13690 34619025 PMC9077724

[25] Mzimela NC Ngubane PS Khathi A. The changes in immune cell concentration during the progression of pre-diabetes to type 2 diabetes in a high-fat high-carbohydrate diet-induced pre-diabetic rat model. Autoimmunity. (2019) 52:27–36. doi: 10.1080/08916934.2019.1575820 30776930

[26] Luvuno M Mbongwa H Khathi A. Development of a novel prediabetes animal model using a high fat high carbohydrate diet: implications for type 2 diabetes. PLoS One. (2017) 13:8–14.

[27] Aronson JK Ferner RE. Biomarkers—a general review. Curr Protoc Pharmacol. (2017) 76:9.23.1–9.23.17. doi: 10.1002/cpph.19 28306150

[28] Luvuno M Mabandla M Khathi A. Voluntary ingestion of a high-fat high-carbohydrate diet: a model for prediabetes. Ponte Int Sci Res J. (2018) 74:74. doi: 10.21506/j.ponte.2018.5.11

[29] Khathi A Serumula MR Myburg RB Van Heerden FR Musabayane CT. Effects of *Syzygium aromaticum*-derived triterpenes on postprandial blood glucose in streptozotocin-induced diabetic rats following carbohydrate challenge. PLoS One. (2013) 8:e81632. doi: 10.1371/journal.pone.0081632, PMID: 24278452 PMC3838356

[30] Gamede M Mabuza L Ngubane P Khathi A. The effects of plant-derived oleanolic acid on selected parameters of glucose homeostasis in a diet-induced pre-diabetic rat model. Molecules. (2018) 23:794. doi: 10.3390/molecules23040794, PMID: 29596390 PMC6017303

[31] Akinnuga AM Siboto A Khumalo B Sibiya NH Ngubane P Khathi A. Bredemolic acid improves cardiovascular function and attenuates endothelial dysfunction in diet-induced prediabetes: effects on selected markers. Cardiovasc Ther. (2020) 2020:1–9. doi: 10.1155/2020/1936406, PMID: 32117470 PMC7036119

[32] Wallace TM Levy JC Matthews DR. Use and abuse of HOMA modeling. Diabetes Care. (2004) 27:1487–95. doi: 10.2337/diacare.27.6.1487 15161807

[33] Friedewald WT Levy RI Fredrickson DS. Estimation of the concentration of low-density lipoprotein cholesterol in plasma, without use of the preparative ultracentrifuge. Clin Chem. (1972) 18:499–502. doi: 10.1093/clinchem/18.6.499, PMID: 4337382

[34] Tabák AG Herder C Rathmann W Brunner EJ Kivimäki M. Prediabetes: a high-risk state for diabetes development. Lancet. (2012) 379:2279–90. doi: 10.1016/S0140-6736(12)60283-9, PMID: 22683128 PMC3891203

[35] Vistisen D Witte DR Brunner EJ Kivimäki M Tabák A Jørgensen ME. Risk of cardiovascular disease and death in individuals with prediabetes defined by different criteria: the Whitehall II study. Diabetes Care. (2018) 41:899–906. doi: 10.2337/dc17-2530, PMID: 29453200 PMC6463620

[36] Mosili P Mkhize BC Ngubane P Sibiya N Khathi A. The dysregulation of the hypothalamic-pituitary-adrenal axis in diet-induced prediabetic male Sprague Dawley rats. Nutr Metab (Lond). (2020) 17:104. doi: 10.1186/s12986-020-00532-1, PMID: 33308255 PMC7731754

[37] Abel ED O'Shea KM Ramasamy R. Insulin resistance: metabolic mechanisms and consequences in the heart. Arterioscler Thromb Vasc Biol. (2012) 32:2068–76. doi: 10.1161/ATVBAHA.111.241984, PMID: 22895668 PMC3646067

[38] Petersen MC Shulman GI. Mechanisms of insulin action and insulin resistance. Physiol Rev. (2018) 98:2133–223. doi: 10.1152/physrev.00063.2017, PMID: 30067154 PMC6170977

[39] Chen L Chen R Wang H Liang F. Mechanisms linking inflammation to insulin resistance. Int J Endocrinol. (2015) 2015:508409:1–9. doi: 10.1155/2015/508409 26136779 PMC4468292

[40] World Health Organization. Use of glycated haemoglobin (HbA1c) in diagnosis of diabetes mellitus: abbreviated report of a WHO consultation. Geneva: World Health Organization (2011). 26158184

[41] Bersoux S Cook CB Wu Q Burritt MF Hernandez JS Verona PM. Hemoglobin A1c testing alone does not sufficiently identify patients with prediabetes. Am J Clin Pathol. (2011) 135:674–7. doi: 10.1309/AJCPJBG0WYRAHN0R, PMID: 21502421

[42] Furman D Campisi J Verdin E Carrera-Bastos P Targ S Franceschi C. Chronic inflammation in the etiology of disease across the life span. Nat Med. (2019) 25:1822–32. doi: 10.1038/s41591-019-0675-0, PMID: 31806905 PMC7147972

[43] Sprague AH Khalil RA. Inflammatory cytokines in vascular dysfunction and vascular disease. Biochem Pharmacol. (2009) 78:539–52. doi: 10.1016/j.bcp.2009.04.029, PMID: 19413999 PMC2730638

[44] Luc K Schramm-Luc A Guzik T Mikolajczyk T. Oxidative stress and inflammatory markers in prediabetes and diabetes. J Physiol Pharmacol. (2019) 70:809–24. doi: 10.26402/jpp.2019.6.01 32084643

[45] Evans JL Goldfine ID. A new road for treating the vascular complications of diabetes: so let's step on the gas. Diabetes. (2016) 65:346–8. doi: 10.2337/dbi15-0029 26798120

[46] Wegner M Araszkiewicz A Piorunska-Stolzmann M Wierusz-Wysocka B Zozulinska-Ziolkiewicz D. Association between IL-6 concentration and diabetes-related variables in DM1 patients with and without microvascular complications. Inflammation. (2013) 36:723–8. doi: 10.1007/s10753-013-9598-y, PMID: 23371411 PMC3648680

[47] Çakar M Balta Ş Şarlak H Akhan M Demirkol S Karaman M. Arterial stiffness and endothelial inflammation in prediabetes and newly diagnosed diabetes patients. Arch Endocrinol Metab. (2015) 59:407–13. doi: 10.1590/2359-3997000000061, PMID: 26201008

[48] Airaksinen K. Silent coronary artery disease in diabetes–a feature of autonomic neuropathy or accelerated atherosclerosis? Diabetologia. (2001) 44:259–66. doi: 10.1007/s001250051609 11270686

[49] Ridker PM. A test in context: high-sensitivity C-reactive protein. J Am Coll Cardiol. (2016) 67:712–23. doi: 10.1016/j.jacc.2015.11.037 26868696

[50] Sarinnapakorn V Wanicagool W. Association between hs-CRP and Hba1c in overweight type 2 diabetic female patients. J Med Assoc Thai. (2013) 96:S54–8. PMID: 23682523

[51] Wang Z Shen X-H Feng W-M Ye G-f Qiu W Li B. Analysis of inflammatory mediators in prediabetes and newly diagnosed type 2 diabetes patients. J Diabetes Res. (2016) 2016:1–10. doi: 10.1155/2016/7965317 PMC4949350 27478850

[52] Katzke VA Sookthai D Johnson T Kühn T Kaaks R. Blood lipids and lipoproteins in relation to incidence and mortality risks for CVD and cancer in the prospective EPIC–Heidelberg cohort. BMC Med. (2017) 15:218. doi: 10.1186/s12916-017-0976-4, PMID: 29254484 PMC5735858

[53] Drew BG Rye K-A Duffy SJ Barter P Kingwell BA. The emerging role of HDL in glucose metabolism. Nat Rev Endocrinol. (2012) 8:237–45. doi: 10.1038/nrendo.2011.235 22271188

[54] Diabetes Canada Clinical Practice Guidelines Expert Committee Punthakee Z Goldenberg R Katz P. Definition, classification and diagnosis of diabetes, prediabetes and metabolic syndrome. Can J Diabetes. (2018) 42:S10–5. doi: 10.1016/j.jcjd.2017.10.003, 29650080

[55] Li N Fu J Koonen DP Kuivenhoven JA Snieder H Hofker MH. Are hypertriglyceridemia and low HDL causal factors in the development of insulin resistance? Atherosclerosis. (2014) 233:130–8. doi: 10.1016/j.atherosclerosis.2013.12.013 24529133

[56] Lee YA Cho EJ Yokozawa T. Effects of proanthocyanidin preparations on hyperlipidemia and other biomarkers in mouse model of type 2 diabetes. J Agric Food Chem. (2008) 56:7781–9. doi: 10.1021/jf800639m, PMID: 18690694

[57] Bhowmik B Siddiquee T Mujumder A Afsana F Ahmed T Mdala IA. Serum lipid profile and its association with diabetes and prediabetes in a rural Bangladeshi population. Int J Environ Res Public Health. (2018) 15:1944. doi: 10.3390/ijerph15091944, PMID: 30200612 PMC6165005

[58] Mabuza LP Gamede MW Maikoo S Booysen IN Ngubane PS Khathi A. Cardioprotective effects of a ruthenium (II) Schiff base complex in diet-induced prediabetic rats. Diabetes Metab Syndr Obes. (2019) 12:217–23. doi: 10.2147/DMSO.S183811, PMID: 30858714 PMC6385740

[59] Marin V Gazzin S Gambaro SE Dal Ben M Calligaris S Anese M. Effects of oral administration of silymarin in a juvenile murine model of non-alcoholic steatohepatitis. Nutrients. (2017) 9:1006. doi: 10.3390/nu9091006, PMID: 28895929 PMC5622766

[60] Huang Q Qin L Dai S Zhang H Pasula S Zhou H. AIP1 suppresses atherosclerosis by limiting hyperlipidemia-induced inflammation and vascular endothelial dysfunction. Arterioscler Thromb Vasc Biol. (2013) 33:795–804. doi: 10.1161/ATVBAHA.113.301220, PMID: 23413429 PMC3637885

[61] Hadjiphilippou S Ray KK. Lipids and lipoproteins in risk prediction. Cardiol Clin. (2018) 36:213–20. doi: 10.1016/j.ccl.2017.12.002 29609750

[62] Rana JS Liu JY Moffet HH Solomon MD Go AS Jaffe MG. Metabolic dyslipidemia and risk of coronary heart disease in 28,318 adults with diabetes mellitus and low-density lipoprotein cholesterol< 100 mg/dl. Am J Cardiol. (2015) 116:1700–4. doi: 10.1016/j.amjcard.2015.08.039, PMID: 26428026

[63] Kilgore M Muntner P Woolley JM Sharma P Bittner V Rosenson RS. Discordance between high non-HDL cholesterol and high LDL-cholesterol among US adults. J Clin Lipidol. (2014) 8:86–93. doi: 10.1016/j.jacl.2013.11.001, PMID: 24528689

[64] Hsu SH-J Jang M-H Torng P-L Su T-C. Positive association between small dense low-density lipoprotein cholesterol concentration and biomarkers of inflammation, thrombosis, and prediabetes in non-diabetic adults. J Atheroscler Thromb. (2018) 275:e253. doi: 10.1016/j.atherosclerosis.2018.06.806 PMC6629751 30587667

[65] Prenner SB Mulvey CK Ferguson JF Rickels MR Bhatt AB Reilly MP. Very low density lipoprotein cholesterol associates with coronary artery calcification in type 2 diabetes beyond circulating levels of triglycerides. Atherosclerosis. (2014) 236:244–50. doi: 10.1016/j.atherosclerosis.2014.07.008, PMID: 25105581 PMC4209900

[66] Eckel RH. What is it about very low density lipoproteins (VLDL) and cardiovascular disease in patients with type 2 diabetes mellitus: is it the triglycerides or the cholesterol? Atherosclerosis. (2014) 237:138–9. doi: 10.1016/j.atherosclerosis.2014.08.048 25238222

[67] Varbo A Benn M Tybjærg-Hansen A Nordestgaard BG. Elevated remnant cholesterol causes both low-grade inflammation and ischemic heart disease, whereas elevated low-density lipoprotein cholesterol causes ischemic heart disease without inflammation. Circulation. (2013) 128:1298–309. doi: 10.1161/CIRCULATIONAHA.113.003008, PMID: 23926208

[68] Verma S Anderson TJ. Fundamentals of endothelial function for the clinical cardiologist. Circulation. (2002) 105:546–9. doi: 10.1161/hc0502.104540, PMID: 11827916

[69] Oak M-H Auger C Belcastro E Park S-H Lee H-H Schini-Kerth VB. Potential mechanisms underlying cardiovascular protection by polyphenols: role of the endothelium. Free Radic Biol Med. (2018) 122:161–70. doi: 10.1016/j.freeradbiomed.2018.03.018, PMID: 29548794

[70] Giannotti G Landmesser U. Endothelial dysfunction as an early sign of atherosclerosis. Herz. (2007) 32:568–72. doi: 10.1007/s00059-007-3073-1 17972030

[71] Kim J Lee H Shin S. Advances in the measurement of red blood cell deformability: a brief review. J Cell Biotechnol. (2015) 1:63–79. doi: 10.3233/JCB-15007

[72] Wasserman DH Wang TJ Brown NJ. The vasculature in prediabetes. Circ Res. (2018) 122:1135–50. doi: 10.1161/CIRCRESAHA.118.311912, PMID: 29650631 PMC5901903

[73] Elgebaly MMEM Kelly A Harris AKHK Elewa H Portik-Dobos V-D Ketsawatsomkron P. Impaired insulin-mediated vasorelaxation in a nonobese model of type 2 diabetes: role of endothelin-1This article is one of a selection of papers published in the special issue (part 1 of 2) on forefronts in endothelin. Can J Physiol Pharmacol. (2008) 86:358–64. doi: 10.1139/Y08-034, PMID: 18516099 PMC3741337

[74] Picchi A Gao X Belmadani S Potter BJ Focardi M Chilian WM. Tumor necrosis factor-α induces endothelial dysfunction in the prediabetic metabolic syndrome. Circ Res. (2006) 99:69–77. doi: 10.1161/01.RES.0000229685.37402.80, PMID: 16741160

[75] Mzimela NC Ngubane PS Khathi A. The Haemolytic changes during progression of pre-diabetes to type 2 diabetes in a high-fat high-carbohydrate diet-induced pre-diabetic rat model. Pak J Nutr. (2021) 20:55–63. doi: 10.3923/pjn.2021.55.63 30776930

[76] Sachidanandam K Hutchinson JR Elgebaly MM Mezzetti EM Wang M-H Ergul A. Differential effects of diet-induced dyslipidemia and hyperglycemia on mesenteric resistance artery structure and function in type 2 diabetes. J Pharmacol Exp Ther. (2009) 328:123–30. doi: 10.1124/jpet.108.142612, PMID: 18941121 PMC2685904

[77] Sud N Black SM. Endothelin-1 impairs nitric oxide signaling in endothelial cells through a protein kinase Cδ-dependent activation of STAT3 and decreased endothelial nitric oxide synthase expression. DNA Cell Biol. (2009) 28:543–53. doi: 10.1089/dna.2009.0865, PMID: 19754268 PMC2880383

[78] Eringa EC Serne EH Meijer RI Schalkwijk CG Houben AJ Stehouwer CD. Endothelial dysfunction in (pre) diabetes: characteristics, causative mechanisms and pathogenic role in type 2 diabetes. Rev Endocr Metab Disord. (2013) 14:39–48. doi: 10.1007/s11154-013-9239-7, PMID: 23417760

[79] Chester AH Yacoub MH. The role of endothelin-1 in pulmonary arterial hypertension. Glob Cardiol Sci Pract. (2014) 2014:62–78. doi: 10.5339/gcsp.2014.29, PMID: 25405182 PMC4220438

[80] Campbell JE Brummel-Ziedins KE Butenas S Mann KG. Cellular regulation of blood coagulation: a model for venous stasis. Blood. (2010) 116:6082–91. doi: 10.1182/blood-2010-01-266395 20864579 PMC3031393

[81] Minors DS. Haemostasis, blood platelets and coagulation. Anaesth Intensive Care Med. (2007) 8:214–6. doi: 10.1016/j.mpaic.2007.02.008

[82] Dahlbäck B. Blood coagulation and its regulation by anticoagulant pathways: genetic pathogenesis of bleeding and thrombotic diseases. J Intern Med. (2005) 257:209–23. doi: 10.1111/j.1365-2796.2004.01444.x, PMID: 15715678

[83] Yun S-H Sim E-H Goh R-Y Park J-I Han J-Y. Platelet activation: the mechanisms and potential biomarkers. Biomed Res Int. (2016) 2016:1–5. doi: 10.1155/2016/9060143, PMID: 27403440 PMC4925965

[84] Chapin JC Hajjar KA. Fibrinolysis and the control of blood coagulation. Blood Rev. (2015) 29:17–24. doi: 10.1016/j.blre.2014.09.003, PMID: 25294122 PMC4314363

[85] Thögersen AM Jansson J-H Boman K Nilsson TK Weinehall L Huhtasaari F. High plasminogen activator inhibitor and tissue plasminogen activator levels in plasma precede a first acute myocardial infarction in both men and women: evidence for the fibrinolytic system as an independent primary risk factor. Circulation. (1998) 98:2241–7. doi: 10.1161/01.CIR.98.21.2241, PMID: 9826309

[86] Binder BR Christ G Gruber F Grubic N Hufnagl P Krebs M. Plasminogen activator inhibitor 1: physiological and pathophysiological roles. Physiology. (2002) 17:56–61. doi: 10.1152/nips.01369.2001 11909993

[87] Festa A D’Agostino R Tracy RP Haffner SM. Elevated levels of acute-phase proteins and plasminogen activator inhibitor-1 predict the development of type 2 diabetes: the insulin resistance atherosclerosis study. Diabetes. (2002) 51:1131–7. doi: 10.2337/diabetes.51.4.1131 11916936

[88] Loscalzo J. Nitric oxide insufficiency, platelet activation, and arterial thrombosis. Circ Res. (2001) 88:756–62. doi: 10.1161/hh0801.089861, PMID: 11325866

[89] Sundaram S Bukowski MR Lie W-R Picklo MJ Yan L. High-fat diets containing different amounts of n3 and n6 polyunsaturated fatty acids modulate inflammatory cytokine production in mice. Lipids. (2016) 51:571–82. doi: 10.1007/s11745-015-4093-x, PMID: 26645280

[90] Kaji H. Adipose tissue-derived plasminogen activator inhibitor-1 function and regulation. Compr Physiol. (2011) 6:1873–96. doi: 10.1002/cphy.c160004 27783862

[91] Shah C Yang G Lee I Bielawski J Hannun YA Samad F. Protection from high fat diet-induced increase in ceramide in mice lacking plasminogen activator inhibitor 1. J Biol Chem. (2008) 283:13538–48. doi: 10.1074/jbc.M709950200, PMID: 18359942 PMC2376236

[92] Mendivil CO Robles-Osorio L Horton ES Hamdy O Caballero AE. Young Hispanics at risk of type 2 diabetes display endothelial activation, subclinical inflammation and alterations of coagulation and fibrinolysis. Diabetol Metab Syndr. (2013) 5:1–8. doi: 10.1186/1758-5996-5-37 23870459 PMC3733973

[93] Ma L-J Mao S-L Taylor KL Kanjanabuch T Guan Y Zhang Y. Prevention of obesity and insulin resistance in mice lacking plasminogen activator inhibitor 1. Diabetes. (2004) 53:336–46. doi: 10.2337/diabetes.53.2.336, PMID: 14747283

[94] Xu L Jiang CQ Lam TH Bao B Cheng KK Thomas GN. Plasminogen activator inhibitor-1 and HbA1c defined prediabetes: the Guangzhou biobank cohort study-CVD. Clin Endocrinol. (2011) 74:528–31. doi: 10.1111/j.1365-2265.2010.03948.x, PMID: 21128994

[95] Palella E Cimino R Pullano SA Fiorillo AS Gulletta E Brunetti A. Laboratory parameters of hemostasis, adhesion molecules, and inflammation in type 2 diabetes mellitus: correlation with glycemic control. Int J Environ Res Public Health. (2020) 17:300. doi: 10.3390/ijerph17010300, PMID: 31906326 PMC6982208

[96] Nkansah C Addai-Mensah O Mensah K Owusu M Ephraim RK Adu P. Plasminogen activator Inhibitor-1 in poorly controlled vs well controlled Type-2 diabetes mellitus patients: a case-control study in a district hospital in Ghana. PLoS One. (2021) 16:e0250090. doi: 10.1371/journal.pone.0250090, PMID: 33857223 PMC8049243

[97] Kahn SE Zinman B Haffner SM O’Neill MC Kravitz BG Yu D. Obesity is a major determinant of the association of C-reactive protein levels and the metabolic syndrome in type 2 diabetes. Diabetes. (2006) 55:2357–64. doi: 10.2337/db06-0116, PMID: 16873701

[98] Basurto L Sánchez L Díaz A Valle M Robledo A Martínez-Murillo C. Differences between metabolically healthy and unhealthy obesity in PAI-1 level: fibrinolysis, body size phenotypes and metabolism. Thromb Res. (2019) 180:110–4. doi: 10.1016/j.thromres.2019.06.013, PMID: 31288156

[99] Kjeldsen SE. Hypertension and cardiovascular risk: general aspects. Pharmacol Res. (2018) 129:95–9. doi: 10.1016/j.phrs.2017.11.003 29127059

[100] Venugopal SK Devaraj S Yuhanna I Shaul P Jialal I. Demonstration that C-reactive protein decreases eNOS expression and bioactivity in human aortic endothelial cells. Circulation. (2002) 106:1439–41. doi: 10.1161/01.CIR.0000033116.22237.F9, PMID: 12234944

[101] Touyz RM. Molecular and cellular mechanisms in vascular injury in hypertension: role of angiotensin II–editorial review. Curr Opin Nephrol Hypertens. (2005) 14:125–31. doi: 10.1097/00041552-200503000-00007, PMID: 15687838

[102] Grassi D Necozione S Lippi C Croce G Valeri L Pasqualetti P. Cocoa reduces blood pressure and insulin resistance and improves endothelium-dependent vasodilation in hypertensives. Hypertension. (2005) 46:398–405. doi: 10.1161/01.HYP.0000174990.46027.70, PMID: 16027246

[103] Jaiswal RK. Endothelin inhibits the atrial natriuretic factor stimulated cGMP production by activating the protein kinase C in rat aortic smooth muscle cells. Biochem Biophys Res Commun. (1992) 182:395–402. doi: 10.1016/S0006-291X(05)80158-5, PMID: 1310017

[104] Kohan DE Rossi NF Inscho EW Pollock DM. Regulation of blood pressure and salt homeostasis by endothelin. Physiol Rev. (2011) 91:1–77. doi: 10.1152/physrev.00060.2009, PMID: 21248162 PMC3236687

[105] Luvuno M Khathi A Mabandla MV. Diet-induced prediabetes: effects of exercise treatment on risk factors for cardiovascular complications. Nutr Metab. (2021) 18:1–9. doi: 10.1186/s12986-021-00573-0 PMC8061036 33888141

[106] Akilen R Pimlott Z Tsiami A Robinson N. Effect of short-term administration of cinnamon on blood pressure in patients with prediabetes and type 2 diabetes. Nutrition. (2013) 29:1192–6. doi: 10.1016/j.nut.2013.03.007, PMID: 23867208

[107] Rathore V Singh N Mahat RK Kocak M Fidan K Ayazoglu T. Risk factors for acute myocardial infarction: a review. Eurasian J Med Invest. (2018) 2:1–7. doi: 10.14744/ejmi.2018.76486

